# Prenatal Manifestation of Transient Abnormal Myelopoiesis: Case Report and Review of the Literature

**DOI:** 10.3390/jcm13164584

**Published:** 2024-08-06

**Authors:** Izabela Walasik, Ewelina Litwińska-Korcz, Monika Szpotańska, Paweł Stanirowski, Aleksandra Księżopolska, Artur Ludwin, Magdalena Litwińska

**Affiliations:** I Department of Obstetrics and Gynecology, Medical University of Warsaw, 02-091 Warsaw, Poland; izabela.a.walasik@gmail.com (I.W.); ewelina.litwinska@gmail.com (E.L.-K.); monika.szpotanska@wum.edu.pl (M.S.); stanirowski@gmail.com (P.S.); axiezopolska@gmail.com (A.K.); ludwin@cm-uj.krakow.pl (A.L.)

**Keywords:** transient abnormal myelopoiesis, transient myeloproliferative disorder, congenital leukemia

## Abstract

**Background**: Congenital malignancies are unusual fetal conditions, and therefore, the data on their prenatal manifestation are limited. Transient abnormal myelopoiesis (TAM) is a hematologic disorder characteristic for babies with trisomy 21 and based on the transient appearance of blast cells in peripheral blood. **Methods**: This paper presents prenatal manifestation of congenital TAM in a newborn with normal karyotype and reviews the literature on prenatal manifestation of this disorder. **Results**: A pregnant woman in her third pregnancy referred herself to the hospital for reduced fetal movements at 30 weeks of gestation. Admission’s ultrasound scan showed an increased middle cerebral artery peak systolic velocity together with hepatomegaly. The patient was admitted to the labor ward for cardiotocography monitoring which showed acute fetal distress with repeated unprovoked decelerations. An emergency cesarean section was conducted and a phenotypically normal female newborn with low Apgar score was delivered. Further examination of the peripheral blood revealed anemia and leukocytosis with high blast proportion. A bone marrow aspirate revealed 70.2% of blasts in a sample with an abnormal karyotype of 47 XX+21. Cytogenetic analysis of the blasts with later microarray comparative genomic hybridization confirmed the presence of GATA1 mutation. However, the buccal smear showed a normal karyotype in the infant. The disease was classified as TAM. **Conclusions**: Our study demonstrates a rare case of prenatal manifestation of TAM in a neonate with a normal karyotype. Obstetricians should pay attention to symptoms like high MCA PSV and hepatosplenomegaly as possible causes of fetal hematological disorders and differentiate it with infection or isoimmunization.

## 1. Introduction

Congenital malignancies are unusual fetal conditions. Considering their rarity, data on prenatal symptoms and prognosis are limited to case reports or small case series. The most common congenital malignancies are neuroblastoma, leukemia, and retinoblastoma [1].

Transient abnormal myelopoiesis (TAM) is a hematologic disorder that occurs in the perinatal period in up to 10% of infants with down syndrome (DS) or mosaic trisomy 21. TAM is characterized by the transient appearance of the blast cells with megakaryoblastic and/or erythroblastic characteristics in the peripheral blood and it is related to GATA1 pathogenic variants [2,3,4,5]. In most cases, TAM resolves spontaneously within the first months of life. However, one in five children who survive the disease will develop another myelodysplastic disease in the future [6].

Prenatal manifestation of hematologic disorders varies from asymptomatic to life-threatening which includes hepatosplenomegaly, hydrops fetalis, as well as intrauterine death [7,8,9,10]. It can be also reflected in easily accessible and routinely assessed fetal dopplers. The middle cerebral artery (MCA) is an important vessel which is commonly used in the assessment of fetal condition. The fetal MCA peak systolic velocity (PSV) is crucial in diagnosing fetal anemia [11]. The common causes of fetal anemia are isoimmunization, infection, fetomaternal hemorrhage, as well as fetal hematological diseases [12].

This paper presents the prenatal manifestation of congenital transient abnormal myelopoesis (TAM) in a newborn with a normal karyotype and reviews the literature on prenatal manifestation of this disorder, emphasizing only one similar case with TAM.

## 2. Materials and Methods

Searches of PubMed, Scopus, Embase, and Web of Science (year 1988) were performed to identify all English language studies that reported prenatal presentation of TAM. Studies were identified using the key words “transient abnormal myelopoesis”, “transient myeloproliferative disorder” and “prenatal manifestation”. We collected only cases with symptoms of TAM described prenatally. The reference lists of retrieved articles were reviewed to locate additional studies. Reviews and articles written in languages other than English were excluded from further analysis.

**Figure 1:**
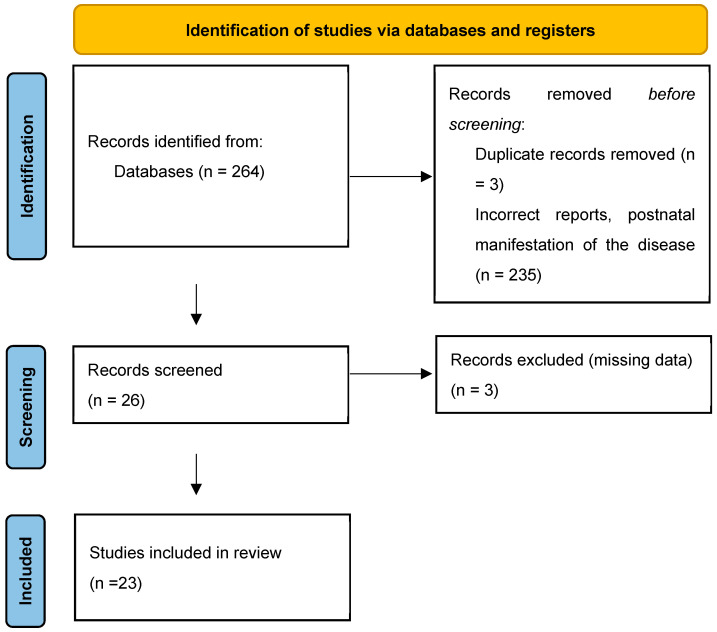
Literature review.

After the initial literature search, publications were analyzed by title and abstract to exclude studies that did not meet the inclusion criteria. A review was undertaken by two independent researchers for articles published between 1988 and 2023 including, firstly, titles and abstracts, and then, full texts of the papers. Following abstract selection, the remaining full-text articles were screened for eligibility. Inclusion criteria included the prenatal manifestation of TAM with or without DS. The exclusion criteria were cases with postnatal manifestation, articles with missing data, and publication other than Polish or English. Agreement before and after discrepancies between researchers were compared (73% vs. 96%) Figure 1.

## 3. Case Presentation

A 29-year-old woman in her third pregnancy, with two previous early miscarriages, referred herself for reduced fetal movements at 30 weeks of gestation. She had gestational diabetes, treated with diet; otherwise, the pregnancy was uneventful. Her routine first trimester scan together with the combined test for aneuploidies showed a low-risk result and the second trimester ultrasound examination showed no fetal abnormalities. The routine third trimester growth scan was scheduled in two weeks. Her blood group was A rhesus positive and no antibodies were detected. At admission’s ultrasound (Voluson E10, GE Healthcare, Chicago, IL, USA), doppler studies showed an increased middle cerebral artery peak systolic velocity (MCA PSV) with the 2.2 multiple of median (MoM). Also, the liver and spleen appeared enlarged. No fetal movements were visualized on the scan. The patient was admitted to the labor ward for cardiotocography monitoring (CTG). CTG (EDAN F9, Guangzhou, China) showed an acute fetal distress with repeated unprovoked decelerations and STV 2.2–3.4 ms. An emergency cesarean section was offered and a female newborn weighing 1600 g with low Apgar score (2/6/6/6 at 1 and 5 min) was delivered. The infant was admitted to the neonatal intensive care unit (NICU) showing difficulties in breathing and incorrect umbilical artery gas analysis (pH 7.12). The first physical examination showed no dysmorphic features. Abdominal palpation revealed enlargement of the liver and spleen.

After admission, blood and urine cultures were taken. An automatic measurement of peripheral blood sample revealed a hemoglobin level of 5.4 g/dL, hematocrit 15.5%, elevated white blood cells 124 − 128 × 10^3^/dL, and normal platelets level. Blood smear analysis showed it was 44% blasts. Based on these results, acute leukemia was suspected. The baby urgently received a red cells transfusion. Because of suspected oncological disease, the patient was transferred to a reference neonatal unit. Subsequent blood analysis showed increased white cell level 161 × 10^3^/uL and biochemical markers of tumor lysis syndrome. Blood smear analysis showed it was 80% blasts. Abdominal ultrasound confirmed hepatosplenomegaly. Antimetabolic agents were administered in order to treat tumor lysis syndrome. A bone marrow aspirate revealed 70.2% blasts in a sample with an abnormal karyotype of 47 XX+21 in a phenotypically normal female neonate. The cytogenetic analysis classified the blasts as FAB M7- megakarioblastic leukemia (AMKL) or TAM. Considering the karyotype of the blast cells and possible diagnosis of Down syndrome (DS), the first line diagnosis was TAM. Knowing that TAM resolves spontaneously in most cases, antimetabolic therapy was discontinued. In order to verify the DS diagnosis, the buccal smear was taken for microarray comparative genomic hybridization (aCGH) (Thermofisher scientific and GATA1 genetic test including three fragments of GATA1 gene (exons 2, 3, 4) which collocated introns using Sanger sequencing (ABI3500). The test was positive for exon 2 GATA1 variant for c.49dupC (p.Gin17ProfsTer23). A test for genetic mosaicism from fibroblasts was not performed.

On the 60th day of life, the female infant was discharged home in a good general condition, hemodynamically stable, feeding with breast milk. Blast cells were no longer detectable.

The child has normal mental and physical development.

## 4. Literature Review

The literature review identified 23 articles that included a total of 43 cases presenting prenatal manifestation of TAM (Table 1). The genetic abnormality that was present in the vast majority of cases was trisomy 21 (42/43 cases).

The diagnosis of TAM was established at a mean gestation age of 31 weeks (23–37 weeks), placing the onset of prenatal manifestation of the disease in the third trimester. A variety of symptoms were identified in the literature. The most common prenatal manifestation of TAM was hepatosplenomegaly (31/43; 72%). Hydrops fetalis was described in 49% of cases (21/43).

Less common prenatal manifestations of TAM included abnormal dopplers indicative of fetal anemia (4/43), polyhydramnios (4/43), oligohydramnios (1/43), and pathologic cardiotocograph (4/43). TAM diagnosed postnatally is defined as a disease that usually resolves spontaneously. However, the presence of prenatal symptoms seems to worsen the prognosis. Among the 43 cases of TAM presenting prenatally, 24 fetuses had fatal outcomes (12 IUD, 12 NND). In three cases, parents opted for a termination of pregnancy. Only 16 fetuses had a normal follow-up. Among the 12 fetuses who died prenatally, the most common symptoms, present in 10 cases, were hepatomegaly and hydrops fetalis. The most common manifestation of TAM among fetuses who died postnatally was hepatomegaly (11/12, 92%). Also, all fetuses with high MCA PSV died before or after birth (4 cases). Three of them had fetal blood sampling which showed anemia, leukocytosis, and provided material for karyotyping. In two cases, intrauterine blood transfusion was performed. In the other two cases, the procedure was abandoned due to acute deterioration of fetal condition and immediate delivery.

The above mentioned data prove that TAM is characteristic for DS fetuses and can manifest with a variety of symptoms. The diagnosis of TAM in a neonate with a normal karyotype is rare. A child with a normal karyotype born with TAM and trisomy 21 in blast cells was described by Dosedla et al. The fetus presented symptoms of mild hepatosplenomegaly, polyhydramnios, and hydrops at 36 weeks of gestation. Fetal dopplers were normal and there was no anemia after birth. Cytogenetic examination with further molecular confirmation of GATA1 gene mutation established the final diagnosis of TAM [31].

## 5. Discussion

This study demonstrates prenatal manifestation of fetal hematopoietic disorder. This is a unique case of TAM in a child with a normal karyotype based on buccal smear and trisomy 21 present in the blast cells only. This study demonstrates prenatal manifestation of TAM with reduced fetal movements, abnormal doppler studies with high MCA PSV, as well as hepatosplenomegaly.

In the presented case, the patient was admitted to the hospital because of reduced fetal movements. Counting fetal movement during pregnancy is a simple method to assess fetal well-being [35]. Periods of decreased fetal activity can be physiological, especially with fetal sleep, and usually last up to 90 min in a full-term fetus [36]. On the other hand, reduced fetal activity is associated with adverse pregnancy outcomes like fetal growth restriction, anemia, infection or even intrauterine fetal death [37,38,39,40]. In the presented case, both admission’s ultrasound and CTG showed abnormal results. It is therefore advisable to perform both CTG and ultrasound whenever pregnant women report reduced fetal activity [41].

In the presented case, high middle cerebral artery peak systolic velocity (MCA PSV) was the main prenatal manifestation of TAM. Middle cerebral artery (MCA) is a vessel commonly used to diagnose fetal anemia [11]. The background of fetal anemia includes isoimmunization, infection, fetomaternal hemorrhage, and, less frequently, fetal hematological diseases [12].

Peripheral blood leukocytosis is a common abnormality described in TAM cases; however, it is impossible to diagnose with ultrasound. While analyzing hemoglobin level in TAM cases, patients are mostly normocythemic, but they can be polycythemic [24] or anemic [7,26]. In the presented case, the red blood cell count after birth was low, which was reflected in high MCA PSV (Hb 5.4 g/dL, hematocrit 15.5%). Fetal anemia led to pathological CTG and prompted immediate delivery.

The literature review (Table 1) on prenatal manifestation of TAM found only four articles describing fetuses with high MCA PSV suggestive of fetal anemia [27,32,33,42]. However, the majority of newborns included in the literature review were found to be anemic after birth without prenatal suspicion of anemia (12/43). Among the fetuses with incorrect MCA blood flow that had fetal blood sampling, hemoglobin level varied from 2.8 to 9.7 g/dL. All fetuses with high MCA PSV reported in the literature died in utero or immediately after birth. In the presented case, the newborn was born in a severe overall condition receiving two points in the Apgar score. It can be, therefore, assumed that the presence of fetal anemia reflected in high MCA PSV is a significant factor that worsens the prognosis. Tamblyn et al., in a systematic review of cases with TAM, suggested that hepatosplenomegaly is a fatal factor for fetuses with TAM, reporting an associated mortality rate of 87.5% (21/24) [43]. Based on our review, together with the case presentation, fetal anemia should be considered as another important risk factor in fetuses with TAM.

According to the literature review (Table 1), hepatosplenomegaly is a common symptom of TAM (31/43 cases). Differential diagnosis of enlarged fetal liver or spleen includes isoimmunization disorders, fetal anemia, cytomegalovirus or parvovirus B19 infection, congestive heart failure, and hepatic tumors [21]. In the presented case, hepatosplenomegaly was a manifestation of TAM. Hepatomegaly in fetuses with TAM results from liver fibrosis, infiltration of blast cells to the liver, as well as extramedullary hematopoiesis [26,42].

In the presented case, the decision to deliver the baby was made based on the pathological indices of CTG which was nonreactive with repeated unprovoked late decelerations and short-term variability of 2.2–3.4 ms. Hartung et al. presented a similar case of a patient at 31 weeks of gestation admitted to the hospital because of reduced fetal movements, reversed end-diastolic flow in the umbilical fetal arteries, and decreased indices in the middle cerebral artery suggestive of the brain-sparing effect. Two days later, an urgent cesarean section was performed due to late decelerations in CTG. The neonate had a genotype of DS with incorrect peripheral blood examination that was later classified as TAM [14]. The presented cases highlight the need for considering fetal hematological disorders when reduced fetal movements are accompanied by hepatosplenomegaly on ultrasound and incorrect CTG.

The postnatal diagnosis of TAM in neonates with a normal karyotype is extremely rare. In order to diagnose this condition, assessment of the total blood count and blast proportion has to be performed. The final diagnosis of TAM is established after cytogenetic analysis and classification of the blast cells with further testing for GATA1 mutation [44,45].

The strength of this study is the sharing of data on symptoms of TAM in a fetus with a normal karyotype. The presented case emphasizes the need to put special attention on the fetal symptoms mentioned above, even if the karyotype is normal based on the initial tests and not to reject TAM diagnosis in a newborn which requires close follow-up for possible development of AML.

The limitations of this study are related to its nature of a case report and the rarity of the condition. Also, fetal blood sampling was not performed since the condition of the fetus prompted urgent delivery. However, the case highlights the importance of performing an ultrasound evaluation upon admission of a patient referred for reduced fetal movements. The examination should include doppler examination also in normally grown fetuses. Another limitation is lack of data on fetal mosaicism. The blood tests usually performed to diagnose TAM are new generation sequencing or whole exome sequencing. These tests are not accurate for detecting newborn mosaicism. The final diagnosis should be based on the tests from newborn fibroblasts as well as analysis of blast cells, which is crucial in post-therapeutic monitoring of AML development.

## 6. Conclusions

Even though the literature reports good prognoses and the self-limiting nature of TAM, fetuses presenting high MCA PSV or hepatosplenomegaly seem to have a fatal outcome. Our study demonstrates the rare manifestation of TAM in a neonate with a normal karyotype and prenatal presentation of hepatosplenomegaly and anemia.

The literature reports leukocytosis as a typical disorder in TAM cases, but this cannot be detected prenatally with noninvasive tolls. Anemia often coexists in TAM cases and is easily detectable by noninvasive MCA doppler examination. This can be an accessory marker for suspected hematological disorders, especially in fetuses presenting hepatosplenomegaly.

Obstetricians should pay attention to symptoms like high MCA PSV and hepatosplenomegaly as possible causes of fetal hematological disorders and differentiate it with infection or isoimmunization. The CTG together with ultrasound examination should be routinely offered if a pregnant woman reports decreased fetal movements. To confirm the TAM diagnosis, it is recommended to perform fetal blood sampling with analysis of the blast karyotype.

### Implication for Clinical Practice

1. High MCA PSV without infection or immunization can be a symptom of TAM in fetuses presenting hepatosplenomegaly.
2. TAM is a possible cause of non-immune hydrops fetalis, fetal anemia, and hepatosplenomegaly.
3. This case illustrates the importance of performing an ultrasound examination that includes doppler studies even if the fetus is growing normally in patients presenting with reduced fetal movements.
4. Do not assume that TAM occurs only in DS neonates.

## Figures and Tables

**Table 1:** Data of 43 fetuses diagnosed with TAM with prenatal symptoms of the disease.

Author	GA (w)	Hydrops Fetalis	Hepatosplenomegaly	Dopplers	Intrauterine Intervention	Birth (w)	Outcome	Fetal Karyotype	Blast Karyotype	Complete Blood Count
Baschat et al., 1998 [13]	26	yes	yes	N/A	N/A	31	IUD	Trisomy 21	Trisomy 21	Anemia, Leukocytosis
Hartung et al., 1988 [14]	31	no	yes	Increased PI UA Decreased PI MCA	N/A	32	Alive	Trisomy 21	N/A	Leukocythosis
Hendricks et al., 1993 [15]	26	yes	no	N/A	C blood transfusion	29	IUD	Trisomy 21	N/A	Anemia
Hendricks et al., 1993 [15]	29	yes	no	N/A	FBS	31	IUD	Trisomy 21	N/A	Leukocytosis Anemia Thrombocytopenia
Macones et al., 1995 [16]	28	yes	yes	N/A	FBS	29	IUD	Trisomy 21	N/A	Leukocytosis Thrombocytopenia
Macones et al., 1995 [16]	30	yes	yes	N/A	FBS	33	Alive	Trisomy 21	N/A	Leukocytosis Anemia
Strobelt et al., 1995 [17]	31	no	yes	N/A	FBS	35	Alive	Trisomy 21	Trisomy 21	Leukocytosis
Siva et al., 1999 [18]	N/A	no	no	N/A	N/A	38	Alive	Trisomy 21	N/A	N/A
Hirashima et al., 2000 [19]	34	no	no	N/A	N/A	35	Alive	Trisomy 21	N/A	N/A
Smercek et al., 2001 [20]	30	yes	yes	UA AEDV	FBS	N/A	IUD	Trisomy 21	Trisomy 21/CD 34+	Leukocytosis Anemia Thrombocytopenia
Smercek et al., 2001 [20]	26	yes	yes	High UA PI	FBS	31	IUD	Trisomy 21	Trisomy 21/CD 34	Leukocytosis Anemia Thrombocytopenia
Smercek et al., 2001 [20]	28	yes	yes	NA	FBS	N/A	IUD	Mosaic Trisomy 21	Trisomy 21	Leukocytosis Anemia Thrombocytopenia
Smercek et al., 2001 [20]	29	yes	yes	N/A	N/A	29	IUD	-	N/A	N/A
Hamada et al., 2001 [21]	35	no	yes	N/A	N/A	36	NND	Trisomy 21	N/A	N/A
Hamada et al., 2001 [21]	35	no	yes	N/A	N/A	38	NND	Trisomy 21	Trisomy 21	N/A
Vimercati et al., 2003 [22]	23	no	yes	N/A	FBS	N/A	TOP	Trisomy 21	N/A	normal
Azancot et al., 2003 [23]	31	no	no	N/A	FBS	32	TOP	Trisomy 21	CD 13, CD 33, CD 34, CD 7	Leukocytosis Thrombocytopenia
Ogawa et al., 2004 [24]	28	no	yes	N/A	N/A	30	Alive	Trisomy 21	N/A	N/A
Ogawa et al., 2004 [24]	32	no	yes	N/A	N/A	39	Alive	Trisomy 21	N/A	N/A
Ogawa et al., 2004 [24]	28	no	no	N/A	N/A	37	Alive	Trisomy 21	N/A	N/A
Kikuchi et al., 2007 [25]	34	no	yes	UA AEDV	N/A	35	NND	Trisomy 21	N/A	N/A
Kikuchi et al., 2007 [25]	37	no	yes	N/A	N/A	38	NND	Mosaic Trisomy 21	N/A	N/A
Kikuchi et al., 2007 [25]	28	no	yes	N/A	N/A	40	Alive	Trisomy 21	N/A	N/A
Kikuchi et al., 2007 [25]	31	no	yes	N/A	N/A	40	Alive	Trisomy 21	N/A	N/A
Hojo et al., 2007 [26]	33	no	no	N/A	FBS	35	Alive	Trisomy 21	N/A	Leukocytosis Thrombocytopenia
Hojo et al., 2007 [26]	28	yes	no	N/A	FBS	32	Alive	Trisomy 21	N/A	Leukocytosis Thrombocytopenia
Hojo et al., 2007 [26]	33	yes	no	N/A	FBS	37	Alive	Trisomy 21	N/A	Leukocytosis Thrombocytopenia
Hojo et al., 2007 [26]	28	yes	yes	N/A	FBS	30	IUD	Trisomy 21	N/A	Leukocytosis Anemia Thrombocytopenia
Hojo et al., 2007 [26]	28	yes	yes	N/A	FBS	31	NND	Trisomy 21	N/A	Leukocytosis Anemia
Hojo et al., 2007 [26]	37	yes	yes	N/A	FBS	37	NND	Trisomy 21	N/A	Leukocytosis Anemia Thrombocytopenia
Hojo et al., 2007 [26]	32	yes	yes	UA AEDV	N/A	32	NND	Trisomy 21	N/A	Leukocytosis Anemia
Gwang et al., 2009 [27]	28	yes	yes	High MCA PSV	FBS	29	IUD	Trisomy 21	N/A	Leukocytosis Anemia Thrombocytopenia
Chen et al., 2009 [28]	32	no	yes	N/A	FBS	36	IUD	46,XX,der(13; 21)(q10;q10),+21 (mosaic trisomy)	N/A	Leukocytosis
Malin et al., 2010 [27]	29	yes	no	MCA PSV high	C blood transfusion	32	NND	Trisomy 21	CD 34 CD 33 CD117 CD 41 CD 42 CD 61 GATA 1 +	Leukocytosis Anemia Thrombocytopenia
Mancuso et al., 2014 [29]	32	yes	yes	N/A	N/A	36	NND	Trisomy 21	N/A	-
Traisrisilp et al., 2016 [30]	31	yes	yes	Pulsation UV	FBS	33	NND	Trisomy 21	N/A	Leukocytosis Anemia Thrombocytopenia
Dosedla et al., 2019 [31]	35	no	yes	N/A	N/A	35	Alive	Normal	GATA 1 +	N/A
Rizzo et al., 2017 [32]	32	no	yes	High MCA PSV	N/A	34	NND	Trisomy 21	GATA 1 +	N/A
Rizzo et al., 2017 [32]	33	no	yes	incorrect	N/A	35	Alive	Trisomy 21	GATA 1 +	N/A
Rizzo et al., 2017 [32]	N/A	no	no	N/A	N/A	39	Alive	Trisomy 21	GATA 1 +	N/A
Muraoka et al., 2022 [33]	30	yes	yes	High MCA PSV	Thoracocentesis	N/A	NND	Trisomy 21	N/A	N/A
Tang et al., 2023 [34]	36	no	yes	N/A	FBS	N/A	IUD	Trisomy 21	GATA 1 +	Leukocytosis Thrombocytopenia
Tang et al., 2023 [34]	36	yes	no	N/A	FBS	N/A	TOP	Trisomy 21	GATA 1 +	Leukocytosis Thrombocytopenia

GA: gestational age; C: cordocentesis; FBS: fetal blood sampling; NND: neonatal death; TOP: termination of pregnancy; IUD: intrauterine death; PI: pulsatility index; UA: umbilical artery; MCA: middle cerebral artery; PSV: peak systolic velocity; AEDV: absent end diastolic flow.

## Data Availability

The datasets used and/or analyzed during the current study are available from the corresponding author on reasonable request.

## References

[1] Sebire N.J., Jauniaux E. (2009). Fetal and placental malignancies: Prenatal diagnosis and management. Ultrasound Obstet. Gynecol..

[2] Taee N., Faraji-Goodarzi M., Safdari M., Bajelan A. (2020). Transient abnormal myelopoiesis in pediatrics with trisomy 21. Clin. Case Rep..

[3] Zipursky A. (2003). Transient leukaemia—A benign form of leukaemia in newborn infants with trisomy 21. Br. J. Haematol..

[4] Chatterjee T., Choudhry V.P. (2013). Childhood myelodysplastic syndrome. Indian J. Pediatr..

[5] Ono R., Hasegawa D., Hirabayashi S., Kamiya T., Yoshida K., Yonekawa S., Ogawa C., Hosoya R., Toki T., Terui K. (2015). Acute megakaryoblastic leukemia with acquired trisomy 21 and GATA1 mutations in phenotypically normal children. Eur. J. Pediatr..

[6] van den Berg H., Hopman A.H., Kraakman K.C., de Jong D. (2004). Spontaneous remission in congenital leukemia is not related to (mosaic) trisomy 21: Case presentation and literature review. Pediatr. Hematol. Oncol..

[7] Robertson M., De Jong G., Mansvelt E. (2003). Prenatal diagnosis of congenital leukemia in a fetus at 25 weeks’ gestation with Down syndrome: Case report and review of the literature. Ultrasound Obstet. Gynecol..

[8] Chelghoum Y., Vey N., Raffoux E., Huguet F., Pigneux A., Witz B., Pautas C., de Botton S., Guyotat D., Lioure B. (2005). Acute leukemia during pregnancy: A report on 37 patients and a review of the literature. Cancer.

[9] Salloum D., Stanirowski P.J., Symonides A., Krajewski P., Bomba-Opoń D., Wielgoś M. (2022). Enlarged Abdominal Lymph Node as a Cause of Polyhydramnios in the Course of Congenital Neonatal Leukaemia: A Case Report and Review of the Literature on Foetal Abdominal Tumours with Coexisting Polyhydramnios. J. Clin. Med..

[10] Foucar K., Friedman K., Llewellyn A., McConnell T., Aisenbrey G., Argubright K., Ballinger L. (1992). Prenatal diagnosis of transient myeloproliferative disorder via percutaneous umbilical blood sampling. Report of two cases in fetuses affected by Down’s syndrome. Am. J. Clin. Pathol..

[11] Mari G., Deter R.L., Carpenter R.L., Rahman F., Zimmerman R., Moise K.J., Dorman K.F., Ludomirsky A., Gonzalez R., Gomez R. (2000). Noninvasive diagnosis by Doppler ultrasonography of fetal anemia due to maternal red-cell alloimmunization. Collaborative Group for Doppler Assessment of the Blood Velocity in Anemic Fetuses. N. Engl. J. Med..

[12] Prefumo F., Fichera A., Fratelli N., Sartori E. (2019). Fetal anemia: Diagnosis and management. Best. Pract. Res. Clin. Obstet. Gynaecol..

[13] Baschat A.A., Wagner T., Malisius R., Gembruch U. (1998). Prenatal diagnosis of a transient myeloproliferative disorder in trisomy 21. Prenat Diagn..

[14] Hartung J., Chaoui R., Wauer R., Bollmann R. (1998). Fetal hepatosplenomegaly: An isolated sonographic sign of trisomy 21 in a case of myeloproliferative disorder. Ultrasound Obstet. Gynecol..

[15] Hendricks S.K., Sorensen T.K., Baker E.R. (1993). Trisomy 21, fetal hydrops, and anemia: Prenatal diagnosis of transient myeloproliferative disorder?. Obstet. Gynecol..

[16] Macones G.A., Johnson A., Tilley D., Wade R., Wapner R. (1995). Fetal hepatosplenomegaly associated with transient myeloproliferative disorder in trisomy 21. Fetal Diagn. Ther..

[17] Strobelt N., Ghidini A., Locatelli A., Vergani P., Mariani S., Biondi A. (1995). Intrauterine diagnosis and management of transient myeloproliferative disorder. Am. J. Perinatol..

[18] Siva S., Smoleniec J. (1999). Antenatal diagnosis of transient abnormal myelopoiesis associated with Down syndrome. Aust. N. Z. J. Obstet. Gynaecol..

[19] Hirashima C., Eguchi Y., Kohmura Y., Minakami H., Sato I. (2000). Isolated pericardial effusion and transient abnormal myelopoiesis in a fetus with Down’s syndrome. J. Obstet. Gynaecol. Res..

[20] Smrcek J.M., Baschat A.A., Germer U., Gloeckner-Hofmann K., Gembruch U. (2001). Fetal hydrops and hepatosplenomegaly in the second half of pregnancy: A sign of myeloproliferative disorder in fetuses with trisomy 21. Ultrasound Obstet. Gynecol..

[21] Hamada H., Yamada N., Watanabe H., Okuno S., Fujiki Y., Kubo T. (2001). Hypoechoic hepatomegaly associated with transient abnormal myelopoiesis provides clues to trisomy 21 in the third-trimester fetus. Ultrasound Obstet. Gynecol..

[22] Vimercati A., Greco P., Gentile A., Ingravallo G., Loverro G., Selvaggi L. (2003). Fetal liver hyperechogenicity on sonography may be a serendipitous sign of a transient myeloproliferating disorder. Prenat. Diagn..

[23] Azancot A., Diehl R., Dorgeret S., Sebag G., Baumann C., Vuillard E., Machado L., Luton D., Oury J.F. (2003). Isolated pericardial effusion in the human fetus: A report of three cases. Prenat. Diagn..

[24] Ogawa M., Hosoya N., Sato A., Tanaka T. (2004). Is the degree of fetal hepatosplenomegaly with transient abnormal myelopoiesis closely related to the postnatal severity of hematological abnormalities in Down syndrome?. Ultrasound Obstet. Gynecol..

[25] Kikuchi A., Tamura N., Ishii K., Takakuwa K., Matsunaga M., Sudo N., Tanaka K. (2007). Four cases of fetal hypoechoic hepatomegaly associated with Trisomy 21 and transient abnormal myelopoiesis. Prenat. Diagn..

[26] Hojo S., Tsukimori K., Kitade S., Nakanami N., Hikino S., Hara T., Wake N. (2007). Prenatal sonographic findings and hematological abnormalities in fetuses with transient abnormal myelopoiesis with Down syndrome. Prenat. Diagn..

[27] Malin G.L., Kilby M.D., Velangi M. (2010). Transient abnormal myelopoiesis associated with Down syndrome presenting as severe hydrops fetalis: A case report. Fetal Diagn. Ther..

[28] Chen C.P., Tsai F.J., Chern S.R., Chang T.Y., Hsu C.Y., Lin H.H., Wang W. (2009). Prenatal diagnosis of 46,XX,DER(13;21)(Q10;Q10),+21 and transient abnormal myelopoiesis in a fetus with hepatosplenomegaly and spontaneous resolution of fetal ascites. Taiwan. J. Obstet. Gynecol..

[29] Mancuso A., Rijhsinghani A. (2014). Elevated delta OD 450 due to transient abnormal myelopoiesis in a Down syndrome fetus with hepatosplenomegaly on ultrasound. Prenat. Diagn..

[30] Traisrisilp K., Charoenkwan P., Tongprasert F., Srisupundit K., Tongsong T. (2016). Hemodynamic assessment of hydrops foetalis secondary to transient myeloproliferative disorder associated with foetal Down syndrome: A case report and literature review. J. Obstet. Gynaecol..

[31] Dosedla E., Turcsanyiova Z., Calda P., Kolenova A. Transient Myeloproliferative Syndrome in Newborn without Down Syndrome Phenotype: A Unique Case Report: Actual Gynecology and Obstetrics, Transient Myeloproliferative Syndrome in Newborn without Down Syndrome Phenotype: A Unique Case Report|Actual Gynecology and Obstetrics. https://www.actualgyn.com/en/article/2019/222.

[32] Rizzo A., Perotti G., Fiandrino G., Spinillo A., Stronati M., Iasci A. (2018). Prenatal diagnosis of transient abnormal myelopoiesis in three fetuses with Down syndrome: Heterogeneous ultrasonographic findings and outcomes. Ultrasound Obstet. Gynecol..

[33] Muraoka J., Yoshimoto N., Ohsawa A., Matsuzawa S., Katsuragi S. (2022). Fetal Distress and Neonatal Death After Thoracoamniotic Shunting Therapy Due to Hydrops Associated with Transient Abnormal Myelopoiesis. Cureus.

[34] Tang H., Hu J., Liu L., Lv L., Lu J., Yang J., Lu J., Chen Z., Yang C., Chen D. (2023). Prenatal diagnosis of Down syndrome combined with transient abnormal myelopoiesis in foetuses with a GATA1 gene variant: Two case reports. Mol. Cytogenet..

[35] Heazell A.E., Frøen J.F. (2008). Methods of fetal movement counting and the detection of fetal compromise. J. Obstet. Gynaecol..

[36] Velazquez M.D., Rayburn W.F. (2002). Antenatal evaluation of the fetus using fetal movement monitoring. Clin. Obstet. Gynecol..

[37] Frøen J.F., Tveit J.V., Saastad E., Børdahl P.E., Stray-Pedersen B., Heazell A.E., Flenady V., Fretts R.C. (2008). Management of decreased fetal movements. Semin. Perinatol..

[38] Levy M., Kovo M., Barda G., Gluck O., Koren L., Bar J., Weiner E. (2020). Reduced fetal movements at term, low-risk pregnancies: Is it associated with adverse pregnancy outcomes? Ten years of experience from a single tertiary center. Arch. Gynecol. Obstet..

[39] Sinha D., Sharma A., Nallaswamy V., Jayagopal N., Bhatti N. (2007). Obstetric outcome in women complaining of reduced fetal movements. J. Obstet. Gynaecol..

[40] Skornick-Rapaport A., Maslovitz S., Kupferminc M., Lessing J.B., Many A. (2011). Proposed management for reduced fetal movements: Five years’ experience in one medical center. J. Matern. Fetal Neonatal Med..

[41] Hofmeyr G.J., Novikova N. (2012). Management of reported decreased fetal movements for improving pregnancy outcomes. Cochrane Database Syst. Rev..

[42] Kim G.J., Lee E.S. (2009). Prenatal diagnosis of transient abnormal myelopoiesis in a Down syndrome fetus. Korean J. Radiol..

[43] Tamblyn J.A., Norton A., Spurgeon L., Donovan V., Bedford Russell A., Bonnici J., Perkins K., Vyas P., Roberts I., Kilby M.D. (2016). Prenatal therapy in transient abnormal myelopoiesis: A systematic review. Arch. Dis. Child. Fetal Neonatal Ed..

[44] Bhatnagar N., Nizery L., Tunstall O., Vyas P., Roberts I. (2016). Transient Abnormal Myelopoiesis and AML in Down Syndrome: An Update. Curr. Hematol. Malig. Rep..

[45] Ohkawa T., Miyamoto S., Sugie M., Tomizawa D., Imai K., Nagasawa M., Morio T., Mizutani S., Takagi M. (2015). Transient abnormal myelopoiesis in non-Down syndrome neonate. Pediatr. Int..

